# Bacteriophage Control of *Pseudomonas* *savastanoi* pv. *glycinea* in Soybean

**DOI:** 10.3390/plants11070938

**Published:** 2022-03-30

**Authors:** Rashit I. Tarakanov, Anna A. Lukianova, Peter V. Evseev, Stepan V. Toshchakov, Eugene E. Kulikov, Alexander N. Ignatov, Konstantin A. Miroshnikov, Fevzi S.-U. Dzhalilov

**Affiliations:** 1 Department of Plant Protection, Russian State Agrarian University—Moscow Timiryazev Agricultural Academy, Timiryazevskaya Str. 49, 127434 Moscow, Russia; tarakanov.rashit@mail.ru (R.I.T.); a.al.lukianova@gmail.com (A.A.L.); an.ignatov@gmail.com (A.N.I.); 2 Shemyakin-Ovchinnikov Institute of Bioorganic Chemistry, Russian Academy of Sciences, Miklukho-Maklaya Str. 16/10, 117997 Moscow, Russia; petevseev@gmail.com; 3 Center for Genome Research, National Research Center “Kurchatov Institute”, Kurchatov Sq. 1, 123098 Moscow, Russia; stepan.toschakov@gmail.com; 4 Research Center of Biotechnology, Winogradsky Institute of Microbiology, Russian Academy of Sciences, Prosp. 60-letia Oktyabrya 7-2, 117312 Moscow, Russia; eumenius@gmail.com; 5 Agrobiotechnology Department, Agrarian and Technological Institute, Peoples Friendship University of Russia (RUDN University), Miklukho-Maklaya Str. 6, 117198 Moscow, Russia

**Keywords:** soybean, bacterial spot, *Pseudomonas savastanoi*, bacteriophage, phage control

## Abstract

Bacterial viruses (bacteriophages) have been considered as potential agents for the biological control of bacterial phytopathogens due to their safety and host specificity*. Pseudomonas savastanoi* pv. *glycinea* (Psg) is a causative agent of the bacterial spotting of soybean (*Glycine max Willd*). The harm caused by this bacterium to crop production and the development of antibiotic resistance in Psg and other pathogenic microorganisms has led to the pursuit of alternative management strategies. In this study, three Psg-specific lytic bacteriophages were isolated from soybean field soil in geographically distant regions of Russia, and their potential for protective action on plants was assessed. Sequencing of phage genomes has revealed their close relatedness and attribution to the genus *Ghunavirus*, subfamily *Studiervirinae*, family *Autographiviridae*. Extensive testing of the biological properties of P421, the representative of the isolated phage group, has demonstrated a relatively broad host range covering closely related *Pseudomonas* species and stability over wide temperature (4–40 °C) and pH (pH 4–7) ranges, as well as stability under ultraviolet irradiation for 30 min. Application of the phages to prevent, and treat, Psg infection of soybean plants confirms that they are promising as biocontrol agents.

## 1. Introduction

Soy (*Glycine max Willd*) is a major staple crop throughout the World. Soybean production is critical for global food safety, particularly as an essential source of vegetable protein for humans and farm animals. Soybeans contain proteins, lipids, carbohydrates and dietary fibre, as well as substantial quantities of vitamins, minerals and phytochemicals [1]. Weed plants, pests and diseases may substantially reduce soybean yield. Bacterial blight is considered most destructive among the diseases of soybean, decreasing the harvest by up to 40% [2]. *Pseudomonas savastanoi* pv. *glycinea* (Coerper, 1919; Gardan et al., 1992) syn—*Pseudomonas syringae* pv. *glycinea* (Coerper, 1919; Young et al., 1978) (Psg) is the causative agent of bacterial blight [1]. The pathogen has been found in 41 countries, covering all climatic zones of soybean production (https://gd.eppo.int/taxon/PSDMGL, accessed on 7 October 2021). Psg was previously attributed to genomospecies 2 of *Pseudomonas syringae* complex and later reclassified as *P. savastanoi* comprising pathovars *savastanoi, glycinea, tabaci* and *phaseolicola* [3]. Currently, the pathogen is represented by ten races recognised by numbers (0–9) dependent on their interaction with the set of ten differentiating host cultivars, of which race 4 is considered to be the most widespread [4,5].

Psg affects all aboveground soy parts, but characteristic symptoms are usually observed on the leaves of middle and upper tiers and on pods. In 5–15 days after infection, necrotic oily spots surrounded by a chlorotic halo appear on the leaves; the spots grow and merge, forming necrotic zones [6]. If the infection occurs early in plant development, it results in dwarfism and the fast death of the plant [7]. The disease is mostly spread through infected seeds [7] or, less frequently, infected weeds and crop residues; this reduces the yield, the oil content of the soybean and the germination of infected seeds [8]. The occurrence of bacterial blight in the field is usually observed in late summer, as a result of a secondary infection in plants [9].

Bacterial disease control includes the use of resistant cultivars, strict crop rotation, the planting of treated seeds only, and the application of bactericides during vegetation. The breeding of resistant soybean varieties is considered to be the most reliable approach, but the development of plant resistance is hindered by the evolution of the virulence of the pathogen. Psg is difficult to control because of its variability and limited knowledge of the pathogen population [8]. Bactericides based on copper compounds, antibiotics and antagonistic bacteria are used for the control of Psg bacterial blight, but the application of antibiotics results in the evolution of resistant bacteria and is restricted in many countries. Copper accumulates in plants and soil and the efficacy of antagonistic bioagents is not high and depends on application conditions [10]. Some biological agents were applied for bacterial blight control on soybean [11].

Bacteriophages (phages) are viruses that specifically infect and lyse bacterial host cells; they are safe for humans and the environment and are often considered as an ecological way to combat pathogenic bacteria, particularly antibiotic-resistant strains [12,13,14], and the properties of phages make them prospective antimicrobials. Some phage-based products are applied commercially against bacterial phytopathogens. Several phages that are effective against Psg have been described in the literature [15,16,17], although in-depth genomic analysis of the phages is missing in these publications and their ex vivo efficacy against the bacterial blight of plants has not been studied. The present research aims to fill some gaps in knowledge of the basic aspects of phage control of soy bacterial blight caused by Psg.

## 2. Results

### 2.1. Bacterial Strains

**Figure 1:**
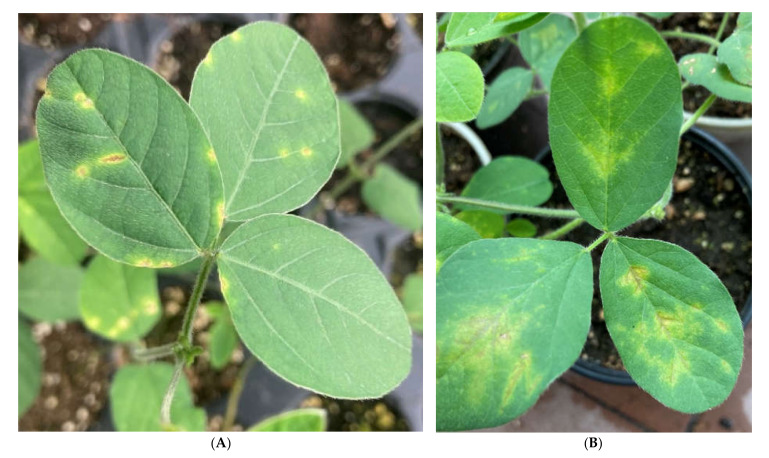
Bacterial blight symptoms on soybean leaves cv. Kasatka 35 days after sowing the inoculated seeds with (**A**)/without (**B**) the phage treatment.

Strain Psg CFBP 2214 was used as a reference for comparison with local isolates of Psg [18]. Samples of soybean plants and seeds with symptoms of bacterial spot, collected in different regions of Russia during the period 2019–2021, were used for bacterial isolation. Over 120 isolates of fluorescent *Pseudomonas* spp. were obtained in total. After microbiological and molecular tests, 12 isolates were selected for further analysis, due to them being the most similar to *Pseudomonas savastanoi* pv. *glycinea* (Supplementary Table S1). All selected strains: (i) were highly virulent for soybean plants after artificial inoculation (Figure 1); (ii) were identical to Psg strain CFBP 2214 in the fluorescence, morphology of colonies on King’s B agar, and in LOPAT test results (+, −, −, −, +); (iii) reacted positively with PCR assay for gene *cfl* (Supplementary Figure S2); (iv) had sequences of gene *cts* fragments that were most similar to the corresponding sequence in the genome of the Psg reference strain ( Figure S1).

### 2.2. Isolation and General Properties of Pseudomonas savastanoi pv. glycinea Bacteriophages

**Figure 2:**
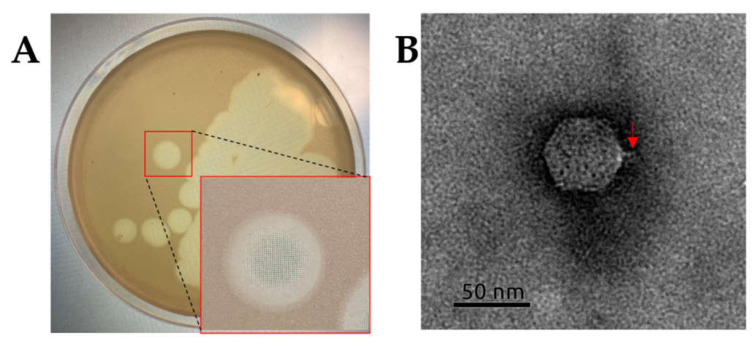
(**A**) Phage plaques shape for P421 on 0.7% upper agar YDC with the host bacterium strain CFBP 2214. Other selected phages presented the same lytic plaque morphology. (**B**) Transmission electron microscopy image of *Pseudomonas* phage P421. The location of the tail is indicated with the red arrow. The scale bar is 50 nm.

All studied phages were isolated from soil under soybean crops in geographically distant regions of soy production in European (Belgorod region, phage P413) and Asian (Amur region, phage P421; Primorsky territory, phage P311) parts of Russia. All three phages were isolated using strain Psg CFBP 2214 for propagation and formed similar large plaques (Ø7–8 mm) with smooth borders (Figure 2A). The activity of isolated phages was tested against a number of *Pseudomonas* spp. and other phytopathogenic bacteria. Phages P421 and P311 infected 13 Psg strains and phage P413 infected ten of the Psg strains tested (Supplementary Table S1). Besides Psg, the phages were infective to some related bacteria: *Pseudomonas syringae* pv. *pisi*, *P. savastanoi* pv. *phaseolicola* and *P. savastanoi* pv. *savastanoi,* and could be considered to be valuable biocontrol agents against a wide range of *P. savastano*i-related pathogens. The phages were avirulent to inoculant *Bradyrhizobium japonicum*, applied for soybean seed treatment, and, thus, were safe for conventional nitrogen fixators.

The transmission electron microscopy image of all three phages (P421 is shown in Figure 2B) demonstrates the typical Podoviral C1 morphology, with an isometric capsid ~58 nm in diameter and a thin ~12 nm-long tail.

**Figure 3:**
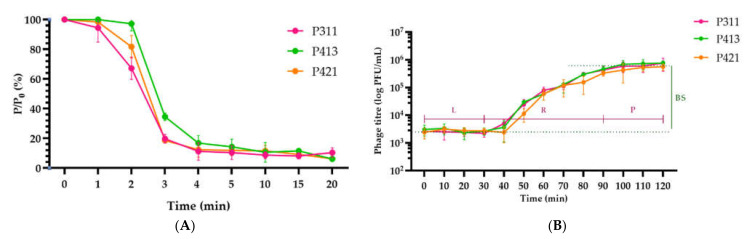
Adsorption curves (**A**) and single-stage phage growth curves (**B**) of phages P311 (pink), P413 (green) and P421 (orange). *P. savastanoi* pv. *glycinea* CFBP 2214 was used as a host. The ordinate axis shows the ratio of the current titer at each time (P) to the original (Po). L–latent phase; R–virion release phase; P–plateau phase; BS–burst size. Values in panels represent the respective mean of three independent trials, respectively, and error bars represent the standard deviation.

The phages adsorbed to Psg host strain CFBP 2214 cells almost completely in 4 min (Figure 3A) at 28 °C. All phages lysed bacteria in ~100 min, forming 230 ± 9 progeny particles per infected bacterial cell (Figure 3B). No phage-resistant bacteria were observed within 3 h of inoculation.

Due to high similarity between the phages, phage P421 was chosen for the further study of Psg control efficacy for *in vitro* and *in planta* greenhouse experiments. Considering a potential application of the phages, a study was undertaken in the context of various environmental factors.

**Figure 4:**
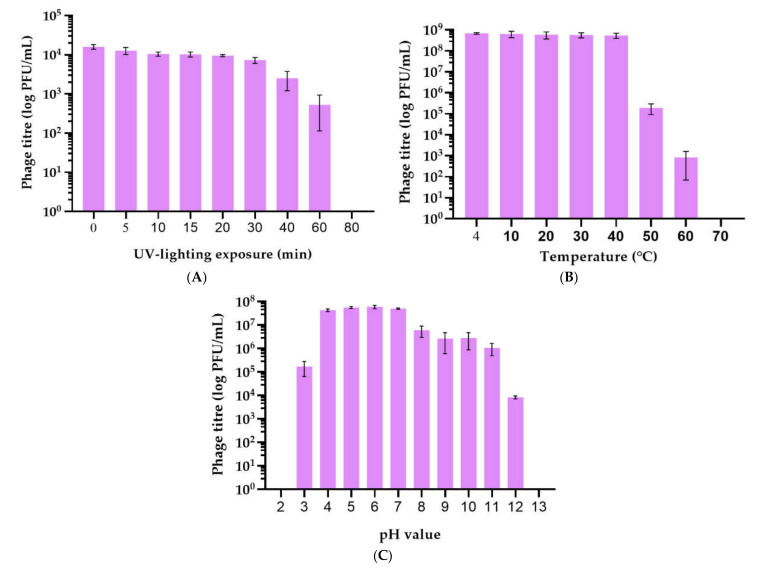
Survival of *Pseudomonas* bacteriophage P421 under different stress factors. The phages were treated with UV irradiation for from 5 to 80 min (**A**), at temperatures from 4 to 70 °C for 1 h (**B**) and with a pH of from 2 to 13 for 1 h (**C**). All tests were carried out three times. Standard deviation (sd) is shown for each bar.

UV (280–315 nm) irradiation decreased phage viability in proportion to the period of treatment (Figure 4A). Complete UV destruction occurred within ~80 min. Phage survival at temperatures from 4 to 70 °C was evaluated. The viability of P421 dropped significantly at temperatures above 40 °C (Figure 4B). In particular, phage suspension with a concentration of 10^7^ PFU/mL lost 90% viability at 50 °C within 1 h. The optimal long storage temperature for the phages was about 4 °C. The phage was stable in SM buffer with a pH from 4 to 7 at 23 °C for 1 h (Figure 4C), but lost viability rapidly at pH 1–2 and pH 12–14.

### 2.3. Phage P421 Genomic and Phylogenetic Characterisation

*Pseudomonas* phages P413 and P421 (GenBank accession numbers #OM282085 and OM256450) have double-stranded DNA genomes of 40,658 and 41,738 base pairs, respectively. The GC-content of the genomes is 56.3% and is close to the GC-content of the host (~58%). Most of the genes of phages P413 and P421 are nearly identical, with occasional indels and nucleotide replacements (Supplementary Tables S2 and S3). The only substantial difference in the structure and gene composition between the genomes is the presence of the gene encoding a minor capsid protein in the P421 genome. The putative minor capsid protein (gene product 37 of P421) attracts attention by the presence of the predicted SGNH hydrolase domain, normally found in tail spike proteins, which might participate in phage adsorption and host recognition [19]; therefore, the genome of P421 was reviewed in greater detail.

**Figure 5:**
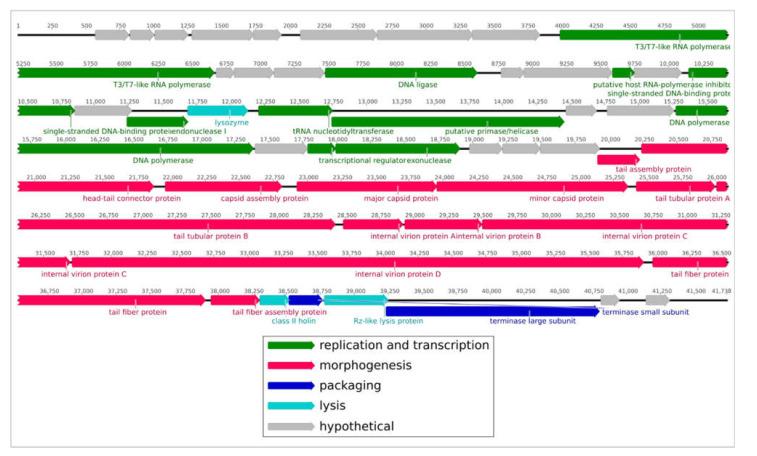
Genetic map of phage P421. The genes are shown as arrowed blocks oriented in the direction of transcription. The nucleotide positions in the genome are shown with numbers above the gene blocks.

The analysis of the P421 genome revealed 51 open reading frames (ORFs) oriented in the forward direction (Supplementary Table S3). The genome contains no tRNA genes. The structure of the genome (Figure 5) is reminiscent of *Escherichia* phage T7 and other *Autographiviridae* bacteriophages. Counting from the 5′-end, the genome includes a block of early genes and a gene of DNA-dependent RNA polymerase. Next, a block of replication and morphogenesis genes comprises the middle gene region, and a block consisting of the terminase and lysis genes constitutes the so-called ‘late gene region’.

**Figure 6:**
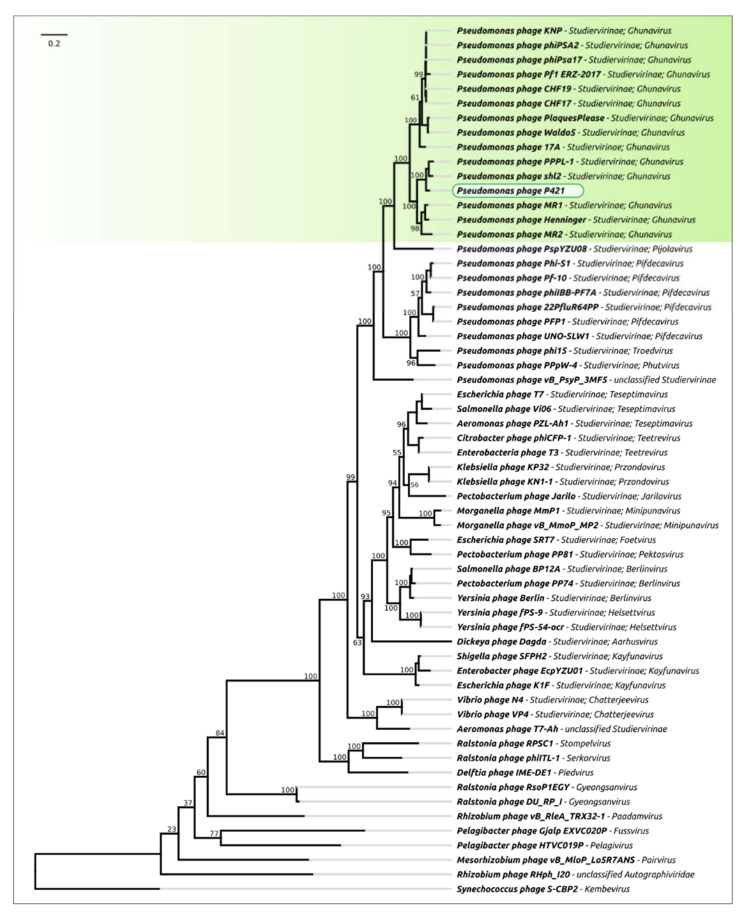
Best-scoring tree found with RAxML, based on the major capsid protein, terminase large subunit, DNA polymerase and RNA polymerase concatenated protein sequences. Taxonomic classification was taken from NCBI sequence attributes and is shown to the right of the phage’s name. Bootstrap support values are shown above their branch as a percentage of 1000 replicates. The scale bar shows 0.2 estimated substitutions per site and the tree was unrooted.

Calculations of average nucleotide identity (ANI) among 15,000 phages deposited in the GenBank phage database as of October 2021 revealed a group of *Pseudomonas* phages clustering with P421 and belonging to the genus *Ghunavirus* of the *Studiervirinae* subfamily, consisting of *sensu lato* T7-like phages (Supplementary Figure S3). The ANI numbers were about 63–92% of those of phage P421. A phylogenetic analysis using concatenated alignments of protein sequences of major capsid protein, a large subunit of terminase, DNA polymerase and RNA polymerase (Figure 6) placed phage P421 together with other *Autographiviridae ghunavirus* phages in a monophyletic group.

### 2.4. Phage Efficacy against Psg Leaf Infection

**Figure 7:**
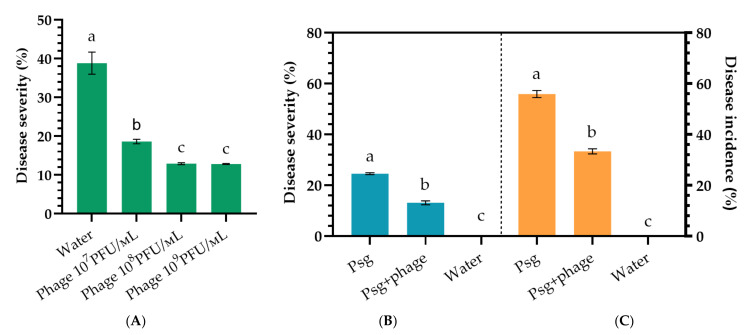
Bacterial blight of soybean caused by artificial inoculation of Psg under phage P421 treatment. (**A**): disease development (severity) on inoculated green plants; (**B**): disease development (severity) after soybean seed inoculation; (**C**): disease development (incidence) after soybean seed inoculation. Values in panels represent the respective mean of three independent trials and error bars represent the standard deviation. Values within columns marked by different letters have significant difference, Duncan’s criteria, *p* = 0.05.

Psg-infected soybean leaves were treated with phage P421 in three repetitions. Disease spread on the leaves was measured using the LeafDoctor program [20] 12 days after phage treatment of previously infected plants. As a result of treatment, disease progress was reduced two-fold compared with the control (Figure 7A). It is intriguing that the greatest efficacy was observed at the phage concentration 10^7^ PFU/mL (52.0%), while disease progress was reduced almost equally after treatments with phage concentrations of 10^8^ PFU/mL (37.6%) and 10^9^ PFU/mL (34.3%).

### 2.5. Phage Efficacy against Psg Seed Infection

Phage P421 treatment of soybean seeds pre-inoculated with Psg displayed a significantly reduced frequency of infected seedlings and disease development rate. The control treatment (using water) showed a rapid development of disease on plants (Figure 7B,C). Due to the daily overhead watering of plants, secondary infection was observed with a severity similar to field outbreak of the disease. The biological efficiency of phage treatment was 59.7% (disease incidence) or 55.0% (disease severity), compared with the control.

## 3. Discussion

In recent decades, the application of bacterial viruses has been considered to be a prospective way to combat bacterial diseases in essential plants [21,22,23]. The successful application of phages against crop diseases is based on the comprehensive characterization of their virulence, physiological and genetic properties.

In this work, an estimate has been made of the suitability of three lytic bacteriophages (P421, P413 and P311) isolated from Psg-contaminated soil samples from geographically remote regions of Russia, most important for soy production: European (Belgorod region) and Asian (Amur region; Primorsky Kray). Soybean is a rather new crop for European Russia, but it was cultivated since ancient time at Amur and Primorsky Kray, close to the main regions of soybean domestication [24]; thus, it is expected that Psg isolates from distant areas in Russia and their corresponding phages possess a high similarity.

All phages displayed lytic activity under a variety of realistic conditions (Figure 4) and demonstrated productive infection for a wide range of target pathogens (Supplementary Table S1). Their revealed properties make them suitable for biocontrol applications.

Phage application in agriculture has many problems, such as resistance to environmental stress and issues such as soil pH, ultraviolet radiation and suboptimal temperature [25,26]. The results presented here show that Psg phages maintained their activity after exposure to a wide range of environmental conditions that occurred during the vegetation of soybeans, except for UV radiation (Figure 4A). The treatment of plants was, however, carried out in the evening and this served as a trigger for the effectiveness of phages on the leaf, taking into account that the period from leaf treatment to exposure to sunlight extended to up to 12 h. During this time, the phages, once on the leaf, were able to destroy some of the Psg bacteria on soybean leaves, causing a significant decrease in the spread and development of the disease. Phytopathogenic Pseudomonads have recently been used extensively for testing the phage control approach. Numerous bacteriophages suitable for therapeutic applications in crop production have been reported [27,28,29,30,31,32] and commercial phage cocktails (Agriphage by Omnilytics (Sandy, UT, USA) against *Xanthomonas campestris* pv. *vesicatoria* and *Pseudomonas syringae* pv. *tomato*) have been offered for plant protection. In recent years, however, the *Pseudomonas* genus, including plant pathogens, has undergone dramatic taxonomic redistribution [33,34] and a number of pathovars have been redefined as new species. The formation of new microbial species is based mostly on genomic properties which are not directly linked to phage susceptibility; therefore, an assessment of the host range should be performed considering the recent taxonomic status of the target pathogen. The phages studied confirmed a broad range of potential host bacteria, including pathovars related to Psg: *Pseudomonas savastonoi*: pv. *phaseolicola*, pv. *savastanoi* and *Pseudomonas syringae* pv. *pisi* virulent for legumes (Supplementary Table S1); thus, the phages possess a broad range of specificity, sufficient for application in the open field. Isolated bacteriophages were found to specifically infect a collection of phytopathogenic Psg acquired from soybean plants in Russia and the type strain of this pathovar, isolated in New Zealand in 1968, whereas non-target pathogenic or environmental bacteria were not affected. RNA-seq analysis of soybeans in Manitoba, Canada, shows a large number of reads and contigs assigned to the bacterial pathogens Psg and other pseudomonads [35], particularly to *P. savastanoi* pv. *phaseolicola* that is most closely related to Psg Pseudomonads [36]; thus, the studied phages can control some other potential soybean pathogens besides Psg.

The particular phages studied were avirulent to other bacteria, including nitrogen-fixing *Rhizobium/Bradyrhizobium* spp., traditionally used for seed inoculation before sowing to enhance nitrogen-fixing nodules on the roots and increase yield [32]. This fact provides scope for the joint application of phages on seeds without an antagonistic relationship to *Bradyrhizobium japonicum* bacteria. Interestingly, none of the phages studied was able to lyse *Pseudomonas fluorescens*, being an epiphyte on plants; thus, the use of these phages has no impact on symbiotic bacteria useful for legumes, as required for a safe biocontrol agent.

Several phages infecting Psg have been previously reported in Refs. [15,16,17], although their description has been limited to their biological properties. Complete genome sequencing is an affordable and useful step for characterising candidate bacteriophages. Besides directly ensuring the absence of genes causing toxicity or integrative features in phage genomes, the correct taxonomic attribution of *de novo* isolated phages enables the scientist to predict unfavourable behaviour typical of a particular phage genus, such as enhanced non-specific transduction. Additional studies might be required to estimate the risk of the preparative use of such phages. Genomic sequences of phages P413 and P421 place them within the genus *Ghunavirus*, subfamily *Studierviriae* (Figure 6). This genus combines podoviral T7-like (*Autographiviridae*, Figure 2) phages related to *P. putida* phage gh-1 [37]. A number of similar phages infecting *P. syringae* [38], *P. fluorescens* [39,40] and *P. aeruginosa* [41] have been studied comprehensively and all of them have demonstrated an obligatory lytic infectional cycle and are considered suitable for formulation as biocontrol agents.

*In vitro* assays of phage activity are usually not enough for a satisfactory assessment of therapeutic potential. The application of bacteriophages against bacterial diseases of seeds and live plants involves many unpredictable factors originating from plant-bacteria and plant–phage interaction; thus, testing the phage protection effect in greenhouse assays provides more practical information. Methods for achieving reproducible infection of soybean plants have been reported, enabling statistically significant estimation of phage application. It is, of course, necessary to continue developing a formulation to increase the stability of phages on leaves [12,42,43]. An assessment of phage effectiveness in the field under real conditions of soybean production should also be investigated and further research is needed to evaluate potential interactions of Psg phages with other agrochemicals commonly used in agriculture, for their joint use in tank mixtures [43].

Nevertheless, the results presented here indicate that phages P413 and P421 have great potential for preventively combatting Psg infections on soybean plants.

## 4. Materials and Methods

### 4.1. Bacterial Strains

Psg strains were obtained from infected soybean leaves and seeds, according to a conventional method [44], with minor modifications. The symptomatic part of the plants/seeds was homogenised in a mortar with the addition of sterile distilled water. Successive ten-fold dilutions of the homogenate were spread on King’s B 1.5% agar medium and incubated at 28 °C for 4–6 days [45]. Typical colonies of fluorescent pseudomonads were purified by subculturing and used for further analysis. The reference strain Psg CFBP 2214 was used in all tests, for comparison. The selected isolates had similar biochemical and morphological characteristics: white, slightly creamy colour of the colonies and round, shiny colonies, which formed siderophore pyoverdin in three days [46] and demonstrated no pectolytic activity. The isolates were tested for LOPAT traits (levan formation, oxidase, pectolytic activity on potato slices, arginine dihydrolase activity and hypersensitivity reaction (microwave) on tobacco plants) [47] and tested with Psg-specific primers.

DNA was isolated from two-day bacterial cultures using a DNA extraction kit “GS-Proba” (AgroDiagnostics, Moscow, Russia) according to the manufacturer’s protocol.

For PCR analysis, primers PsgFOR-1 (′5-GGC GCT CCC TCG CAC TT-3′) and PsgREV-2 (′5-GGT ATT GGC GGG GGT GC-3′) (product size—650 bp) were used; they are specific for the *cfl* gene encoding coronafacate ligase, required for the synthesis of the phytotoxin coronatin [6].

For amplification, a 25 µL of PCR mixture contained 5 µL of 5× Master Mix (5× MasDDTaqMIX-2025, Dialat, Moscow, Russia); 1.0 µL of each primer with a concentration of 10 pM and 5 ng of target DNA. PCR amplification was performed in a T100 thermal cycler (Bio-Rad, Hercules, CA, USA) according to the recommended programme [48].

Amplicons were separated by electrophoresis in 1.5% agarose gel stained with ethidium bromide in a 0.5× TBE buffer and analysed in Gel Doc XR+ (Bio-Rad).

The pathogenicity of isolates was tested on 25-day-old soybean plants cv. Kasatka grown in 0.5-L plastic pots with a peat and perlite mixture (Veltorf, Velikie Luki, Russia). Plants were grown in a greenhouse at a temperature of 25/20 °C (day/night) in natural light. A suspension of bacterial cells of a two-day pathogen culture with a concentration of 10^6^ CFU/mL was applied for inoculation by rubbing the leaves with a cotton swab soaked in the suspension [44]. CFBP strain 2214 was used as a positive control and sterile water was used as a negative control. Three plants were infected for each strain. Disease evaluation was carried out after eight days, when typical symptoms appeared. The experiment was carried out three times.

For a more detailed genetic characterisation of the isolated strains, the sequencing of the citrate synthase (*cts*) gene was performed using primers cts-Fs (5′-CCCGTCGAG CTGCCAATWCTGA-3′) and cts-Rs (5′-ATCTCGCACGGS GTRTTGAACATC-3′); 25 µL of PCR mixture containing 5 µL of 5× Master Mix (5× MasDDTaqMIX-2025, Dialat.); 1.0 µL of each primer with a concentration of 10 pM; 5 ng of target DNA—5 µL; PCR-grade water—13 µL. DNA was amplified according to [49] and sequenced by Evrogen (Moscow, Russia).

The evolutionary history was inferred using the Minimum Evolution method [50]. The optimal tree is shown (Supplementary Figure S2). The percentage of replicate trees in which the associated taxa clustered together in the bootstrap test (500 replicates) is shown next to the branches [51]. The evolutionary distances were computed using the Maximum Composite Likelihood method [52] and are expressed as the number of base substitutions per site. The ME tree was searched using the Close-Neighbour-Interchange (CNI) algorithm [53] at a search level of 1. The Neighbour-joining algorithm [54] was used to generate the initial tree. This analysis involved 42 nucleotide sequences. Codon positions included were 1st + 2nd + 3rd + Noncoding. All ambiguous positions were removed for each sequence pair (using the pairwise deletion option). There were 531 positions in the final dataset. Evolutionary analyses were conducted in MEGA X [55].

### 4.2. Bacteriophage Isolation, Propagation and Purification

Bacteriophages specific to *P. savastanoi* pv *glycinea* were isolated from soil samples taken from fields with soybean bacterial blight. Geographical coordinates of sampling spots were in the Far East 50°10′18″ N, 128°00′19″ E (Amur region, Russia, phage P421), 44°07′37″ N, 132°01′04″ E (Primorsky territory, Russia, phage P311) and Central black soil region 51°10′04″ N, 37°49′51″ E (Belgorod region, Russia, phage P413).

Phages were propagated using Psg strain CFBP 2214 at 28 °C according to a previously published protocol [56]. Phage lysate was treated with chloroform and bacterial debris was pelleted by centrifugation at 8000× *g* for 20 min, followed by filtration of the supernatants through 0.22 µm pore-size membrane filters, (Millex-GV, Millipore, Cork, Ireland) and the addition of DNAse I (0.5 mg/mL, 1 h). Phage filtrates were concentrated by ultracentrifugation at 100,000× *g* at 4 °C for 2 h, using a Beckman SW28 rotor. Further phage purification was performed by ultracentrifugation in CsCl step gradient (0.5–1.7 g/mL) at 22,000× g for 2 h; opalescent band was collected and dialysed against SM buffer (10 mM Tris-HCl, pH 7.5, 10 mM MgSO_4_, 100 mM NaCl) and phage suspension was stored at 4 °C.

### 4.3. Electron Microscopy

Negative-staining transmission electron microscopy [57] was performed to assess the morphology of isolated Psg phages. Aliquots of the purified phages were loaded onto a carbon-coated copper grid, subjected to glow-discharge and then negatively stained with 1% uranyl acetate for 30 s and air-dried. Prepared grids were examined using a JEM-2100 200 kV transmission electron microscope (JEOL, Tokyo, Japan). The dimensions of each phage were averaged among ~20 individually measured particles.

### 4.4. Determination of Phage Host Specificity

The host specificity of phages P311, P413 and P421 was tested against a number of collection strains representing Psg, other pathovars of *P. savastanoi*, phytopathogenic *Pseudomonas* spp. and other bacteria (Supplementary Table S1) using the double-layer agar method [58]. For this, 300 µL of bacterial cultures grown in YD medium (YDC without CaCO_2_) at 28 °C to OD_600_ ~0.3 were mixed with 4 mL of soft agar (YD supplemented with 0.6% agarose). The mixtures were plated onto the nutrient agar. Then, the phage suspension (~10^6^ plaque-forming units (PFU) per mL) was spotted on the soft agar lawns and incubated at 28 °C for 18–24 h. Strains of *Bradyrhizobium japonicum* were obtained from commercial preparations used for soybean seed inoculation (Supplementary Table S1) by culturing them on selective medium BJSM [59]. The isolates were then purified through three rounds of subculturing and pure isolates used for trials.

### 4.5. Phage Adsorption and One-Step Growth Experiments

A sample of phage P421 was inoculated in a growing culture of Psg strain CFBP 2214 at the approximate MOI of 0.1 and incubated at room temperature. A volume of 100 µL of samples was taken every minute and mixed with 850 µL of SM buffer with 50 µL of chloroform. After centrifugation, the supernatants were titered for further determination of unabsorbed phages using the plaque assay method [60]. The procedure was carried out three times.

For the one-step growth experiments, exponentially growing culture of Psg CFBP 2214 (OD_600_ ~0.2 (~10^4^ CFU/mL)) was infected with phage P421 at a MOI of 0.1. Then, the mixture was incubated at 28 °C, aliquots were collected every 10 min, chilled, pelleted by centrifugation (21,000× *g*, 3 min, 4 °C) and the supernatant was titered using YD agar. Plaques were counted after overnight incubation at 28 °C. The procedure was carried out in triplicate and the results were averaged. The latent period was determined as the interval between the adsorption of the phages to the bacterial cells and the release of phage progeny. The burst size of the phage was determined as the ratio of the average number of free phage particles after the release phase (plateau average [PFU/mL]), to their number during the latency phase (latent average [PFU/mL]).

### 4.6. Phage Stability under Different Conditions

The ability of phages to survive in different environmental conditions was assessed (i) by incubation of the phage sample (10^6^ PFU/mL in SM buffer) at 4, 10, 20, 30, 40, 50, 60 and 70 °C for one h in a ThermoMixer F 2.0 (Eppendorf, Hamburg, Germany); (ii) by the addition of a range of buffer solutions (20 mM Tris-HCl/20 mM Na citrate/20 mM Na phosphate), adjusted with NaOH to a pH in the range 2–12 to a 10^7^ PFU/mL phage, with further incubation at 25 °C for one h; (iii) by the exposure of a phage sample (10^6^ PFU/mL in SM buffer) to UV-B (280–315 nm) radiation using a PL-S9W/12/2p lamp (Philips, Amsterdam, The Netherlands) according to [42]. Treated phages were titrated on YD agar/CFBP 2214 using the plaque assay method. Procedures were carried out in triplicate and the results were averaged.

### 4.7. Genome Sequencing and Annotation

Phage DNA was isolated from concentrated and purified high titer phage stock using the standard phenol-chloroform method after incubation of the sample in 0.5% SDS and 50 µg/mL proteinase K at 65 °C for 20 min.

Fragment genome libraries were prepared using 200 ng of phage genomic DNA as a starting material. DNA was fragmented by ultrasound, using an ME220 focused ultrasonicator (Covaris, Woburn, MA, USA) with the following parameters: iterations—7; duration—10 s; peak power—50; duty factor—20%; cycles per burst—1000. Fragmented DNA was used as an input for library preparation using the NEBNext Ultra II DNA Library Prep Kit for Illumina (New England Biolabs, Ipswich, MA, USA) according to the manufacturer’s instructions. The library was sequenced using an MiSeq sequencer (Illumina, San Diego, CA, USA), using 2 * 250 bp paired-end chemistry, resulting in approximately 435,000 read pairs.

The generated reads were assembled *de novo* into single contig using SPAdes v. 3.11.1 [61] with default parameters.

The phage genome was annotated by predicting and validating open reading frames (ORFs) using Prodigal 2.6.1 [62] and Glimmer 3.02 [63]. ORFs identified were manually curated to ensure fidelity. Functions were assigned to ORFs using a BLAST search on NCBI databases HHpred [64] and Phyre2 [65]. tRNA coding regions were checked with Prokka [66]. Resulting genomes were visualised using Geneious Prime version 2021.1.0 [67].

Annotated genomes of phages P413 and P421 were deposited to NCBI GenBank under accession numbers OM 285085 and OM 256450, respectively.

### 4.8. Phylogeny and Taxonomy Studies

Phage genomes for comparison were downloaded from GenBank. Genes of terminase large subunits were extracted from the annotated genomes. Protein alignments were made using MAFFT (L-INS-i algorithm, BLOSUM62 scoring matrix, 1.53 gap open penalty, 0.123 offset value) [68]. Phylograms were generated based on the amino acid sequences of proteins and their concatenated alignments. The best protein models were found with MEGA X [55]. Trees were constructed using the maximum likelihood (ML) method with an RAxML program [69] and an LG protein model [70]. The robustness of the trees was assessed for RAxML by fast bootstrapping (1000). Average nucleotide identity (ANI) was computed using the OrthoANIu tool, with default settings [71], and the VIRIDIC server [72].

### 4.9. Phage Control of Bacterial Blight on Soybean

Experiments on the artificial infection of seeds and plants and phage control were conducted in the period May–August 2021.

#### 4.9.1. Artificial Latent Infection of Soybean Seeds

The artificial latent infection of soybean seeds proceeded according to Ref. [73], with some modifications. A three-day culture of Psg strain CFBP 2214 was grown at 18 °C on King’s B agarised medium [74]. Bacteria were resuspended in sterile 10 mM MgCl_2_ to OD_600_ ~0.2 (~10^4^ CFU/mL). Seeds of soybean cv. Kasatka were washed in 75% ethanol for 2 min, then ethanol was decanted and aqueous 50% bleach/0.002% Tween 20 (*v*/*v*) was added and gently mixed for 8–10 min. The seeds were washed in distilled H_2_O to remove bleach and left in a moist chamber for 2 h to enable them to swell. Swollen seeds were perforated with a sterile toothpick in two places each and placed into a conical flask. Bacterial suspension was added to cover the seeds and the flask was placed in a vaccuum chamber at −10^5^ Pa for 10 min. Treated seeds were dried on paper towels to remove excessive liquid.

#### 4.9.2. Phage Application on Seeds

Bacteriophages were applied to infected soybean seeds by mixing (13 mL of suspension in water per 1 kg of seeds) and the seeds were planted in the turf/perlite substrate (Veltorf, Velikie Luki, Russia) in 40-cell plastic transplant trays (cell volume 0.12 L, AgrofloraPack, Vologda, Russia). Seeds covered with phages were obtained by mixing seeds and a solution containing phages with a certain concentration. Three experiments (with phage concentrations of 10^7^, 10^8^ and 10^9^ PFU/mL) and a control (using water spraying) were treated, in triplicate, with 40 seeds each.

#### 4.9.3. Artificial Infection of Soybean Leaves

Psg infection of vegetating soybean cv. Kasatka plants was achieved by bacterial infiltration into the soybean leaf using 1113 AirControl airbrush (JAS, Ningbo, China) according to Ref. [75], with modifications. A bacterial suspension was prepared as for seed infection, with the addition of wetting agent Silwet Gold (Chemtura, Philadelphia, PA, USA) to a concentration 0.01% (*v*/*v*). Ternary leaves of plants were infiltrated on a V2 stage pressing the leaf to a flat surface (Petri dish) to avoid pressure damage from the airbrush spray. All leaves were infiltrated with an average dose of 7 mL suspension, with a concentration of 10^9^ PFU/mL per ternary leaf. The negative control was sprayed with an equivalent amount of water, using a wetting agent. The plants were grown in 0.5 L plastic pots on a turf/perlite substrate, in a greenhouse, at an average temperature day/night of 25/20 °C and with natural illumination, without fertilisers. Watering was carried out daily by sprinkling. Two days before, and 24 h after, inoculation, relative humidity was maintained at ~95% at a constant temperature of 27 °C.

#### 4.9.4. Phage Application on Leaves

Phage treatment on vegetating plants was carried out using 25-day soybean plants, 12 h before bacterial inoculation. Then, 5 mL of phage suspension was applied to each plant with an airbrush, to complete the wetting of the leaves. Further cultivation of plants proceeded as mentioned previously. Four variants (control (water) and phage concentrations 10^7^, 10^8^ and 10^9^ PFU/mL) were studied using 3 repetitions of 20 plants each.

The incidence rate was recorded as the percentage of plants that showed symptoms on their leaves. An assessment of the development of the disease, in terms of the infection of adult plants, was carried out on the 12th day after infection, using the LeafDoctor application (https://www.quantitative-plant.org/software/leaf-doctor, accessed on 21 October 2021) installed on an iPhone SE 2. All leaves from all plants were individually photographed and analysed by moving the threshold slider until only symptomatic tissues were transformed into a blue shade and calculating the percentage of diseased tissue according to the developer’s recommendations [20,76]. In the experiment with seed treatment, similar calculations were carried out, but after the V3 stage had been reached (i.e., 35 days after sowing).

Statistical processing of the analysed data was carried out using the method of variance analysis, with Statistica 12.0 (StatSoft, TIBCO, Palo Alto, CA, USA), comparing averages according to the Duncan criterion. Percentage data were converted to arcsines before processing. Plots were created with GraphPad Prism 9.2.0.

## Data Availability

Annotated genomic sequences of *Pseudomonas* phages P413 and P421 have been deposited to NCBI GenBank and are available under accession numbers OM 282085 and OM 256450, respectively.

## Supplementary Materials

The following are available online at https://www.mdpi.com/article/10.3390/plants11070938/s1: Table S1: The spectrum of lytic activity of phages against strains *Pseudomonas savastanoi* pv. *glycinea* and related species. Table S2: Annotation of *Pseudomonas* phage P413 genome. Table S3: Annotation of *Pseudomonas* phage P421 genome. Figure S1: Phylogenetic tree of nucleotide sequences of the *Pseudomonas* spp citrate synthase (*gltA*) gene. Orange frames indicate the strains that have been characterised. Figure S2: PCR detection of the *cfl* gene. M: 100+ bp DNA Ladder (Evrogen) Molecular Weight Marker; K-: control (reaction without DNA); G1–G17—analysed Psg strains. Figure S3: VIRIDIC generated heatmap of *Pseudomonas* phages P413, P421 and related phages. The colour coding indicates the clustering of the phage genomes based on intergenomic similarity. The numbers represent the similarity values for each genome pair, rounded to the first decimal.
