# Supplementary material for: Bacteriophage Control of Pseudomonas savastanoi pv. glycinea in Soybean
Source: Plants (Basel). 2022 Mar 30;11(7):938. doi: 10.3390/plants11070938 (PMC9003214; doi:10.3390/plants11070938)
Supplement: Supplementary file 1 [file plants-11-00938-s001.zip › plants-1642256-supplementary.pdf]

plants

MDPI

Article

Bacteriophage Control of *Pseudomonas savastanoi* pv. *glycinea* in Soybean

Rashit I. Tarakanov [1], Anna A. Lukianova [1,2], Peter V. Evseev [2], Stepan V. Toshchakov [3], Eugene E. Kulikov [4], Alexander N. Ignatov [1,5], Konstantin A. Miroshnikov [1,2]*, and Fevzi S.-U. Dzhalilov [1]*

Supplementary Materials

Table S1. The spectrum of lytic activity of phages against strains *Pseudomonas savastanoi* pv. *glycinea* and related species.

**Bacterial species**	**Strain**	**Origin, host, year of Isolation**		**Source**	**Spot formation for phage:**		
					311	P421	413
Pseudomonas savastanoi pv. *glycinea*	G1	Russia, *Glycine max*	Voronezh region, 2019	This work	+	+	+
	G2	Russia, *Glycine max*	Amur region, 2019		-	+	+
	G3	Russia, *Glycine max*	Amur region, 2020		+	+	+
	G4	Russia, *Glycine max*	Khabarovsk krai, 2020		+	+	+
	G5	Russia, *Glycine max*	Voronezh region, 2020		+	+	+
	G6	Russia, *Glycine max*	Voronezh region, 2021		+	+	+
	G7	Russia, *Glycine max*	Amur region, 2021		+	+	+
	G8	Russia, *Glycine max*	Primorskiy krai, 2021		+	+	+
	G9	Russia, *Glycine max*	Khabarovsk krai, 2021		+	+	+
	G10	Russia, *Glycine max*	Amur region, 2021		+	+	-
	G11	Russia, *Glycine max*	Khabarovsk krai, 2021		+	+	-
	G17	Russia, *Glycine max*			+	-	-
	2214	New Zealand, *Glycine max*, 1968		CFBP[a]	+	+	+
Pseudomonas syringae pv. *pisi*	2105	New Zealand, *Pisum sativum*, 1969		CFBP	+	+	+
Pseudomonas savastanoi pv. *phaseolicola*	1429	Netherlands, *Phaseolus vulgaris*, 1973		CFBP	+	+	+
Pseudomonas savastanoi pv. *savastanoi*	1342	USA, *Olea europaea*, 1962		NCPPB[b]	+	+	+
Pseudomonas syringae pv. *syringae*	10604 [ATCC 19310, ICMP 3023, ICPB	UK, *Syringa vulgaris*, 1972		DSM[c]	-	-	-

	PS255, NCPPB 281]					
Pseudomonas syringae pv. *aptata*	2-04	Russia, *Beta vulgaris*, 2017	RSAU-MTAA[e]	-	-	-
Pseudomonas putida	24	Russia, soil, 2017	RSAU-MTAA	-	-	-
Pseudomonas asplenii	7231	Japan, *Oryza sativa*	DSM	-	-	-
Pseudomonas fluorescens	grape ROOT 4	Russia, soil, 2012	RSAU-MTAA	-	-	-
Xanthomonas axonopodis pv. *phaseoli*	2534	USA, *Phaseolus vulgaris*, 1986	CFBP	-	-	-
Xanthamonas glycines	Xgly	Russia, *Glycine max* , 1960	RSAU-MTAA	-	-	-
Xanthomonas phaseoli v. *sojense*	Xphs	Russia, *Glycine max* , 1960	RSAU-MTAA	-	-	-
Curtobacterium flaccumfaciens pv. *flaccumfaciens*	3418	Hongrie, *Phaseolus vulgaris*, 1957	CFBP	-	-	-
Xanthamonas campestris pv. *campestris*	NCPPB528T [ICMP 13, LMG 568, PDDCC 13] (ATCC 33913)	UK, Brussels sprout, *Brassica oleracea* var. *gemmifera*, 1958	NCPPB	-	-	-
Bradyrhizobium japonicum	Semia 5079, Semia 5080 and Semia 532 C	Commercial inoculants Atuva and Haykout Super[d]	-	-	-	-

+ lysis of bacterial cells (spot formation), − lack of bacterial cell lysis (no spot formation).

a-CFBP – CIRM-CFBP: International Center for Microbial Resources, Collection for Plant-associated Bacteria, French.

b-NCPPB – National Collection of Plant Pathogenic Bacteria, United Kingdom.

c-DSM-Leibniz Institute DSMZ-German Collection of Microorganisms and Cell Cultures.

d- commercial inoculants for processing soybean seeds and stimulating the formation of rhizobial nodules. Registered in Russia under the brands Atuva (Syngenta LLC) and Haikout Super (BASF LLC).

e- RSAU-MTAA –microorganisms collection of Russian State Agrarian University-Moscow Timiryazev Agriculture Academy.

Table S2. Genomic annotation for *Pseudomonas* phage P413

ORF	Gene product	Start	End	Length	Direction
1	hypothetical protein	571	819	249	forward
2	hypothetical protein	826	1005	180	forward
3	hypothetical protein	1008	1256	249	forward
4	hypothetical protein	1280	1735	456	forward
5	hypothetical protein	1732	1938	207	forward
6	hypothetical protein	2079	2645	567	forward
7	hypothetical protein	2642	3346	705	forward
8	hypothetical protein	3343	3840	498	forward
9	T3/T7-like RNA polymerase	3985	6663	2679	forward
10	hypothetical protein	6675	6812	138	forward
11	hypothetical protein	6809	7102	294	forward
12	hypothetical protein	7102	7473	372	forward
13	DNA ligase	7478	8596	1119	forward
14	hypothetical protein	8770	8943	174	forward
15	hypothetical protein	8940	9590	651	forward
16	putative host RNA-polymerase inhibitor	9587	9751	165	forward
17	hypothetical protein	9748	10098	351	forward
18	single-stranded DNA-binding protein	10148	10855	708	forward
19	hypothetical protein	10855	11277	423	forward
20	endonuclease I	11238	11687	450	forward
21	lysozyme	11687	12127	441	forward
22	tRNA nucleotidyltransferase	12207	12752	546	forward
23	putative primase/helicase	12742	14451	1710	forward
24	hypothetical protein	14465	14689	225	forward
25	hypothetical protein	14769	15260	492	forward
26	DNA polymerase	15273	17387	2115	forward
27	hypothetical protein	17398	17787	390	forward
28	transcriptional regulator	17784	17993	210	forward
29	exonuclease	17990	18907	918	forward
30	hypothetical protein	18975	19217	243	forward
31	hypothetical protein	19220	19498	279	forward
32	hypothetical protein	19495	19932	438	forward
33	tail assembly protein	19916	20227	312	forward
34	head-tail connector protein	20239	21873	1635	forward
35	capsid assembly protein	21958	22815	858	forward
36	major capsid protein	22925	23953	1029	forward
37	tail tubular protein A	24319	24900	582	forward
38	tail tubular protein B	24910	27327	2418	forward

39	internal virion protein A	27384	27824	441	forward
40	internal virion protein B	27836	28405	570	forward
41	internal virion protein C	28406	30598	2193	forward
42	internal virion protein D	30609	34811	4203	forward
43	tail fiber protein	34875	36809	1935	forward
44	tail fiber assembly protein	36847	37206	360	forward
45	class II holin	37206	37424	219	forward
46	terminase small subunit	37421	37669	249	forward
47	Rz-like lysis protein	37682	38152	471	forward
48	terminase large subunit	38133	39869	1737	forward
49	hypothetical protein	40064	40237	174	forward

Table S3. Genomic annotation for *Pseudomonas* phage P421

ORF	Gene product	Minimum	Maximum	Length	Direction	locus_tag
1	hypothetical protein	571	819	249	forward	PF_00001
2	hypothetical protein	826	1005	180	forward	PF_00002
3	hypothetical protein	1008	1256	249	forward	PF_00003
4	hypothetical protein	1280	1735	456	forward	PF_00004
5	hypothetical protein	1732	1938	207	forward	PF_00005
6	hypothetical protein	2079	2645	567	forward	PF_00006
7	hypothetical protein	2642	3346	705	forward	PF_00007
8	hypothetical protein	3343	3840	498	forward	PF_00008
9	T3/T7-like RNA polymerase	3985	6663	2679	forward	PF_00009
10	hypothetical protein	6675	6812	138	forward	PF_00010
11	hypothetical protein	6809	7102	294	forward	PF_00011
12	hypothetical protein	7102	7473	372	forward	PF_00012
13	DNA ligase	7478	8596	1119	forward	PF_00013
14	hypothetical protein	8770	8943	174	forward	PF_00014
15	hypothetical protein	8940	9590	651	forward	PF_00015
16	putative host RNA-polymerase inhibitor	9587	9751	165	forward	PF_00016
17	hypothetical protein	9748	10098	351	forward	PF_00017
18	single-stranded DNA-binding protein	10148	10855	708	forward	PF_00018
19	hypothetical protein	10855	11277	423	forward	PF_00019
20	endonuclease I	11238	11687	450	forward	PF_00020
21	lysozyme	11687	12127	441	forward	PF_00021
22	tRNA nucleotidyltransferase	12207	12752	546	forward	PF_00022
23	putative primase/helicase	12742	14451	1710	forward	PF_00023
24	hypothetical protein	14465	14689	225	forward	PF_00024
25	hypothetical protein	14769	15260	492	forward	PF_00025
26	DNA polymerase	15273	17387	2115	forward	PF_00026

27	hypothetical protein	17398	17787	390	forward	PF_00027
28	transcriptional regulator	17784	17993	210	forward	PF_00028
29	exonuclease	17990	18907	918	forward	PF_00029
30	hypothetical protein	18975	19217	243	forward	PF_00030
31	hypothetical protein	19220	19498	279	forward	PF_00031
32	hypothetical protein	19495	19932	438	forward	PF_00032
33	tail assembly protein	19916	20227	312	forward	PF_00033
34	head-tail connector protein	20239	21873	1635	forward	PF_00034
35	capsid assembly protein	21958	22815	858	forward	PF_00035
36	major capsid protein	22925	23953	1029	forward	PF_00036
37	minor capsid protein	23953	25359	1407	forward	PF_00037
38	tail tubular protein A	25418	25999	582	forward	PF_00038
39	tail tubular protein B	26009	28426	2418	forward	PF_00039
40	internal virion protein A	28483	28923	441	forward	PF_00040
41	internal virion protein B	28935	29504	570	forward	PF_00041
42	internal virion protein C	29505	31697	2193	forward	PF_00042
43	internal virion protein D	31708	35910	4203	forward	PF_00043
44	tail fiber protein	35974	37908	1935	forward	PF_00044
45	tail fiber assembly protein	37946	38305	360	forward	PF_00045
46	class II holin	38305	38523	219	forward	PF_00046
47	terminase small subunit	38520	38768	249	forward	PF_00047
48	Rz-like lysis protein	38781	39251	471	forward	PF_00048
49	terminase large subunit	39232	40800	1569	forward	PF_00049
50	hypothetical protein	40812	40949	138	forward	PF_00050
51	hypothetical protein	41144	41317	174	forward	PF_00051

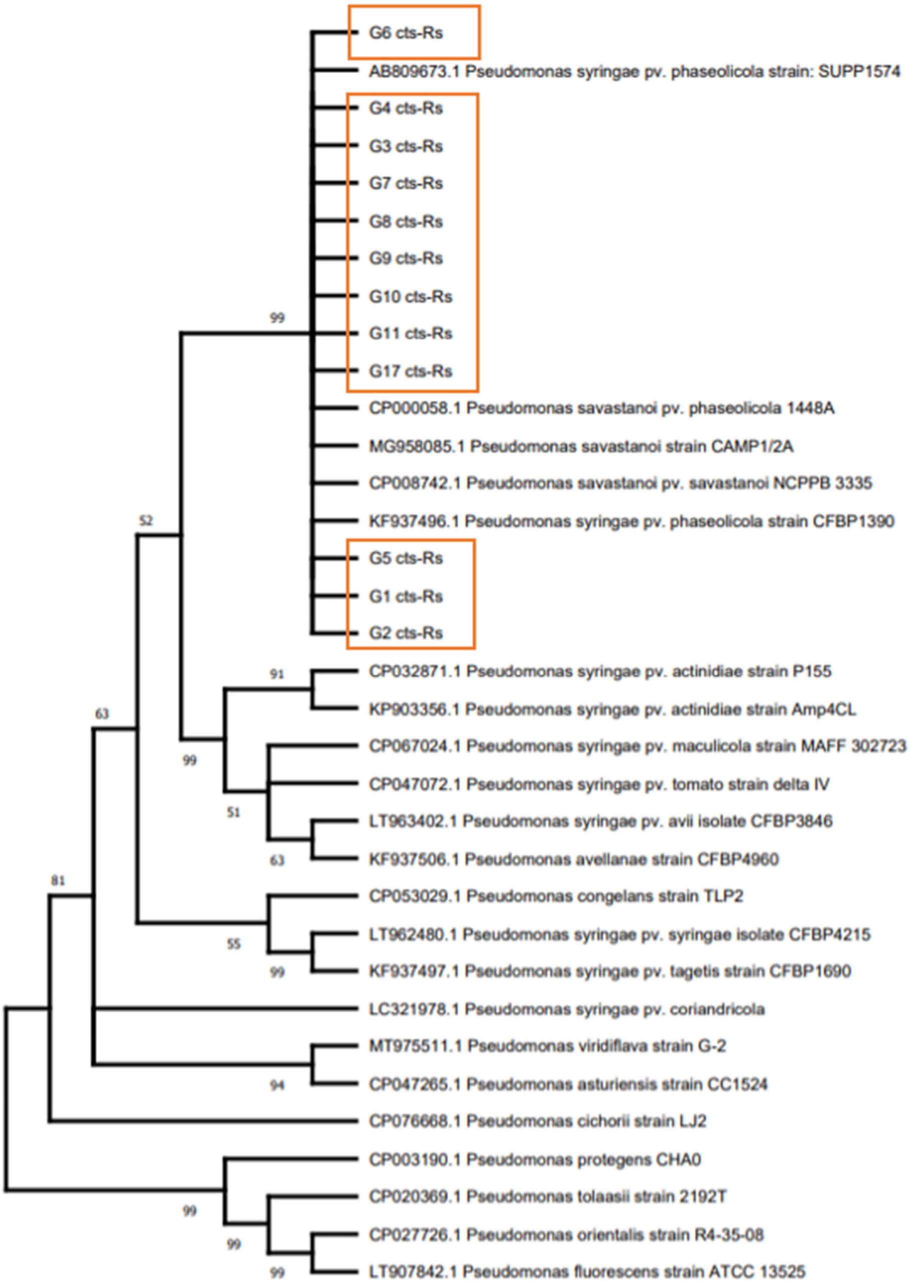

Figure S1. Phylogenetic tree of nucleotide sequences of the *Pseudomonas* spp citrate synthase (*gltA*) gene. Orange frames are circled by the strains we have characterized.

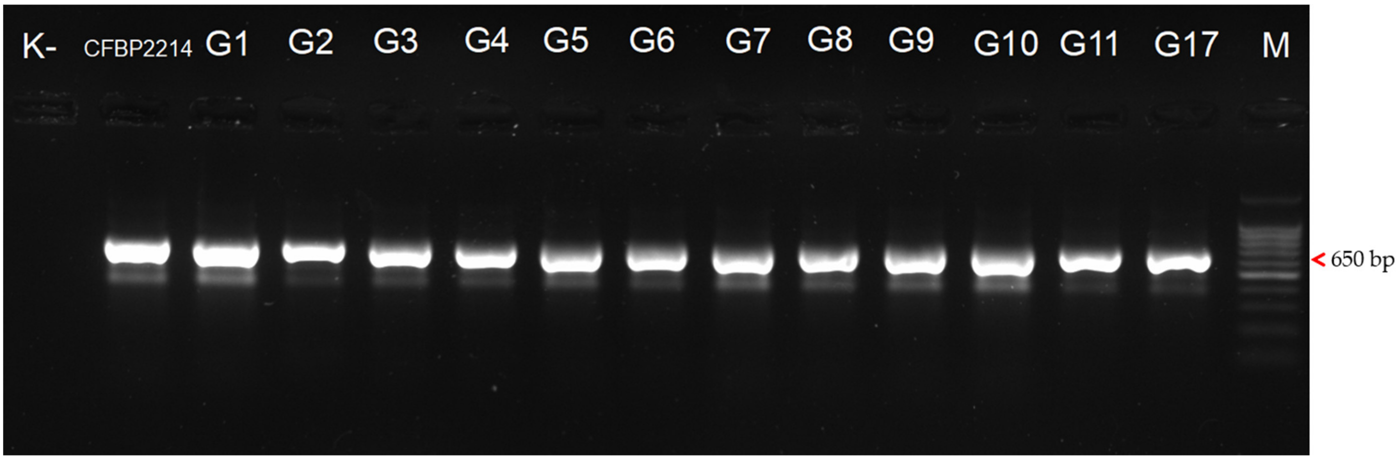

Figure S2. PCR detection of the *cfl* gene. M: 100+ bp DNA Ladder (Evrogen) Molecular Weight Marker (#NL002); K-: control (reaction without DNA); G1-G17 – analysing strains Psg.

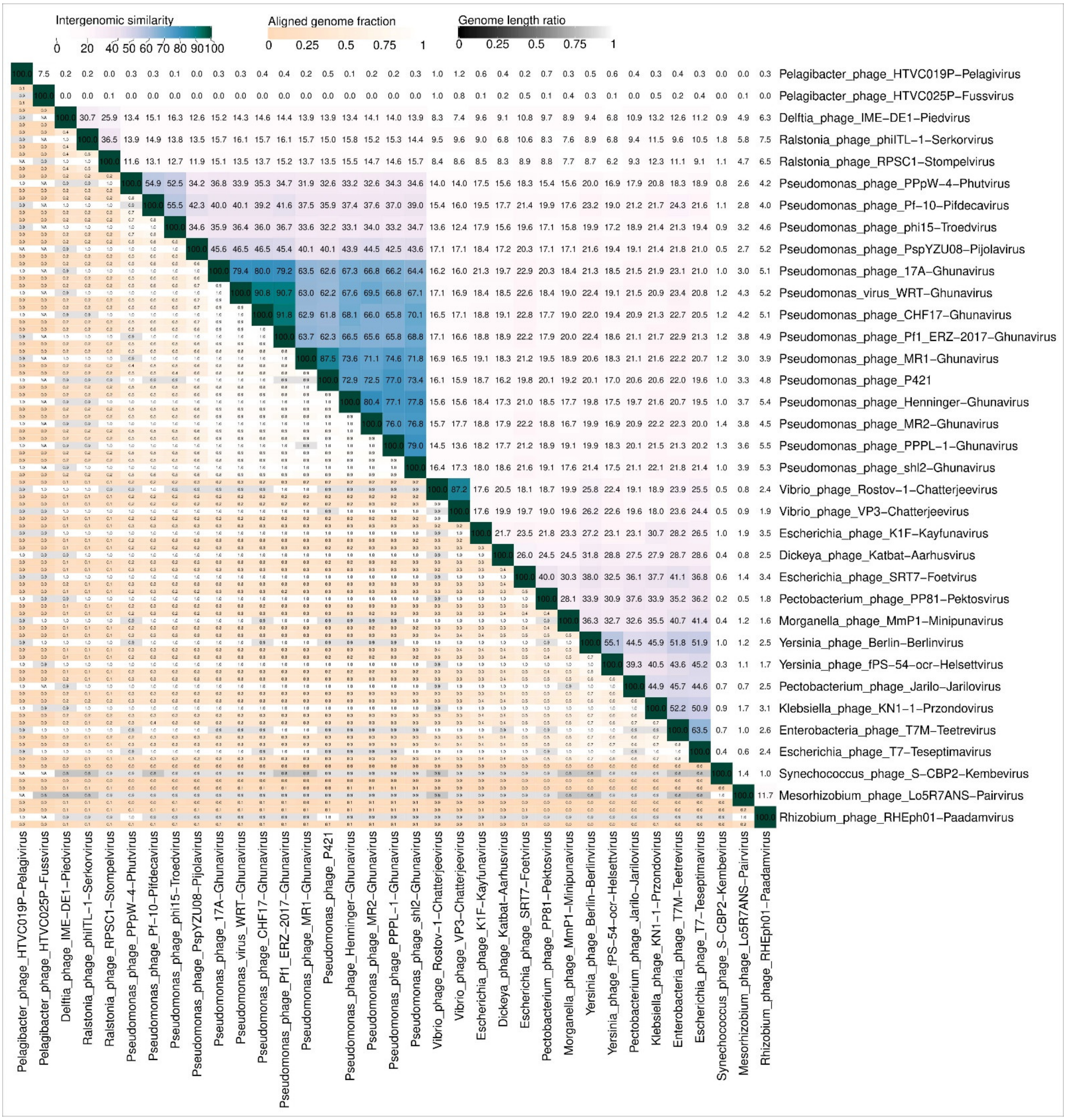

Figure S3. VIRIDIC generated heatmap of *Pseudomonas* phages P413, P421, and related phages. The colour coding indicates the clustering of the phage genomes based on intergenomic similarity. The numbers represent the similarity values for each genome pair, rounded to the first decimal.
